# Integration of unilateral tooth mucosa-supported retentive surgical guide design in implant guided surgery: an in-vitro study

**DOI:** 10.1186/s12903-025-07277-4

**Published:** 2025-12-11

**Authors:** Esraa A. Elnadoury, Yousria S. Gaweesh, Shaimaa M. Abu el Sadat, Mervat E. Abd-Ellah

**Affiliations:** 1 Department of Oral Medicine, Oral Periodontology, Oral Diagnosis and Oral radiology Faculty of Dentistry, Alexandria University, Champollion Street, Alexandria, Azarita 002034868066 Egypt; 2 Department of Oral Medicine, Oral Periodontology, Oral Diagnosis and Oral Radiology, Faculty of Dentistry, Alexandria University, Alexandria, Egypt; 3 Department of Oral and Maxillofacial Radiology, Faculty of Dentistry, Ain-Shams University, Cairo, Egypt; 4 Department of Prosthodontics, Faculty of Dentistry, Alexandria University, Alexandria, Egypt

**Keywords:** CBCT, Implant, Partially edentulous, Surgical guide, Stability

## Abstract

**Purpose:**

This study aimed to compare a retentive surgical guide design with a fixation pin design in unilateral mandibular distal extension cases and evaluate their resulting implant deviations.

**Materials and methods:**

Ten epoxy models with a soft tissue-simulating layer were used, each fitted with two surgical guide designs. The retentive guide featured clasp-like extensions, a 0.07 mm guide-to-tooth offset, and a 2.3 mm thickness. The fixation pin design had a 0.2 mm offset, a 3 mm thickness, and one fixation pin in the distal edentulous region. Eight directional forces were applied through the drill handle. The models were scanned before and after force application. Simulated implants were inserted into 180 scans, resulting in a total of 306 implants. Post-scan data were superimposed onto the initial plan to assess implant and guide deviation.

**Results:**

The baseline deviation was 0.34 ± 0.19 mm for the retentive design and 0.30 ± 0.14 mm for the fixation pin design (*P* = 1.00). Both designs produced simulated implant deviations within the recommended 2 mm safety margin. However, the retentive design showed significantly greater vertical implant deviation compared to the fixation design (0.99 ± 0.76 mm vs. 0.50 ± 0.34 mm, *P* < 0.001).

**Conclusion:**

Incorporating retention features into surgical guides reduces simulated implant displacement in unilateral distal extension cases. However, the retentive guide exhibited larger implant deviations than the fixation design and required additional software adjustments, indicating a need for further refinement.

## Introduction

The literature states different classifications and designs of implant surgical guides. Surgical guides are classified based on the degree of guidance, the incorporation of sleeves, the sleeve design, the manufacturing technique, and the type of surgical exposure [1–4]. They are classified according to their support as tooth-supported, bone-supported, mucosa-supported, bone-mucosa-supported, and tooth-mucosa-supported. Prior research confirmed that tooth-supported guides are superior to mucosa- and bone-supported guides [5–7].

Several factors influence the accuracy of surgical guides [5, 8–10]. Among the most critical are positioning errors and intrinsic inaccuracies introduced during the design and fabrication process [8, 11].

A greater deviation in implant placement was observed when applying unilateral tooth-supported guides, compared to bilateral tooth-supported guides. This is thought to be primarily due to guide instability, which is more pronounced in the mandible due to its reduced surface area compared to the maxilla [6, 12]. Therefore, additional means of retention are required in mandibular unilateral tooth-supported guide designs.

Various methodologies were evaluated in the literature to enhance guide retention, including increasing guide extension, incorporating supporting teeth undercuts, adding bone anchorage screws in distal extensions, utilizing guide stabilizers, changing the support pattern of the surgical guide from tooth support to tooth-mucosa support, and using fixation pins [13–17]. Incorporating fixation pins into the design of surgical guides has been proven to improve guide stability, assist initial positioning, and reduce implant deviation [18–23]. They also eliminate the need for extensive guide extensions, which may compromise access, increase cost, and minimize patient comfort [16]. However, there is a lack of studies comparing guide fixation against other means of retention.

A guide-tooth offset of 0.1 to 0.2 mm is generally recommended to compensate for inaccuracies associated with CBCT imaging, optical scanning, and the superimposition process [5, 10, 24]. Nevertheless, the application of the recommended offset in the literature was presumed to participate in the initial guide deviation. A recent design for a retentive surgical guide was proposed, featuring a reduced guide-teeth offset, decreased guide thickness, and clasp-like components to enhance force resistance, minimize guide movement, and increase stiffness. This design was reported to improve the accuracy of bilateral tooth-supported surgical guides within single-tooth replacement cases [24].

Kobe et al. [24] argued that by examining the retentive surgical guide using the recommended methodology in the literature, the smallest displacements could not be recorded [25–28]^.^ Therefore, they recommended a new methodology to detect surgical guide displacement and the resulting implant deviation against standardized forces through simulated implant placement [24].

This in vitro study aimed to evaluate a modified tooth-mucosa-supported retentive surgical guide design and guide with a fixation pin in mandibular distal extension situations and their resulting implant deviation using Kobe et al. [24]’s method. The current study's null hypothesis assumes no statistically significant difference between the two surgical guide designs.

## Material and methods

This in vitro study was conducted following the approval of the ethical community at the Faculty of Dentistry, Alexandria University (International NO: 00010556-IORG0008839). It was prepared following CRIS guidelines for reporting in vitro studies [29]. The sample size was according to Kobe et al. [24]’s results, adopting a power of 80% (= 0.20) to detect a standardized effect size in implant vertical deviation (primary outcome) of 0.405 and a level of significance of 5% (α error accepted = 0.05) [30]. The sample size was calculated using GPower version 3.1.9.2 [31]. The calculated sample consisted of 20 guides incorporating 306 implants.

This in vitro study used epoxy resin models with soft tissue-replicating material of mandibular unilateral distal extension cases (Kennedy class II) indicated for implant placement with missing premolars and molars. A uniform thickness of flexible polyurethane soft tissue simulation layer was applied over the epoxy resin model to mimic the resilience of oral mucosa [32]. Models were scanned using an extraoral scanner (Cerec InEos X5, Sirona). The model’s CBCTs were taken with the following parameters:120 kVp, 5 mA, and 26.9 s at 0.25 mm voxel size via an I-CAT Next Generation machine (Imaging Sciences International). The Standard Tessellation Language (STL) files with Digital Imaging and Communications in Medicine (DICOM) data of the models were imported into BlueSky software (BlueSky-Plan 4; BlueSky Bio) for surgical guide planning.

**Fig. 1:**
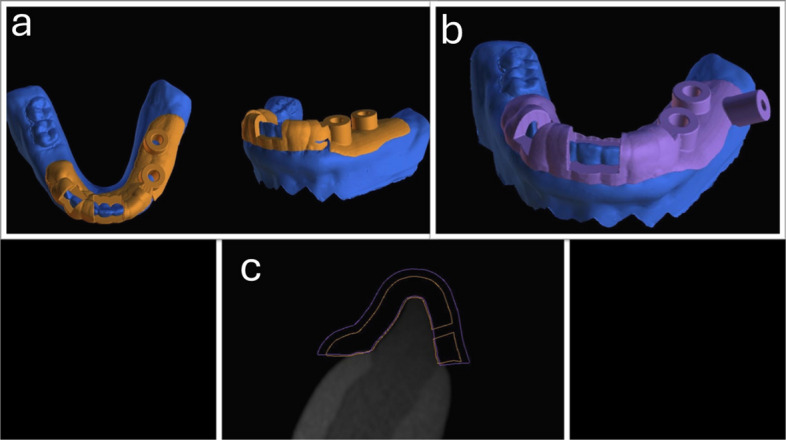
Surgical guide designs. **a** Retentive design, **b** Fixation design, **c** Showing the difference in thickness and guide tooth offset between the two designs, with the orange retentive design and purple fixation design

Two unilateral tooth-mucosa-supported surgical guides were created for each model, with two implants planned in the first premolar and first molar location with a standard size (Neodent®, 10 mm in length; Ø 4 mm). Corresponding sleeves were generated following the manufacturer's recommendations for the guided kit. For the retentive design (Fig. 1A), the predetermined software settings were altered. The guide-teeth offset was reduced to 0.07 mm, and a 2.3 mm guide thickness was used. Some alterations were made to the original design by Kobe et al. [24] since their design was used in bounded tooth-supported situations. Two modified buccal marginal parts of the guide, similar to a retentive clasp, were designed on both sides. The surgical guide crossed the midline and extended to the premolar area on the opposite side, involving six or more teeth [17].

The fixation design (Fig. 1B) was fabricated with the predetermined parameters in BlueSky bio software (a guide to tooth offset of 0.20 mm and a thickness of 3 mm). The same extension as the retentive design was applied. In the distal edentulous area, a fixation screw was planned with corresponding sleeves measuring 3.1 mm in diameter and 7.5 mm in height [16, 20].

Surgical guides were printed by a stereolithography (SLA) printer (Form 3, Formlabs Inc., Somerville, MA, USA) with the clear Surgical Guide Resin V1 (Formlabs Inc., Somerville, MA, USA). The basic printing-support generation was selected using a 0.65 density, a 0.30-mm touchpoint size, and a 0.1 mm layer thickness, with the horizontal orientation of the surgical guides [33]. The post-curing technique was applied following the manufacturer's instructions.

**Fig. 2:**
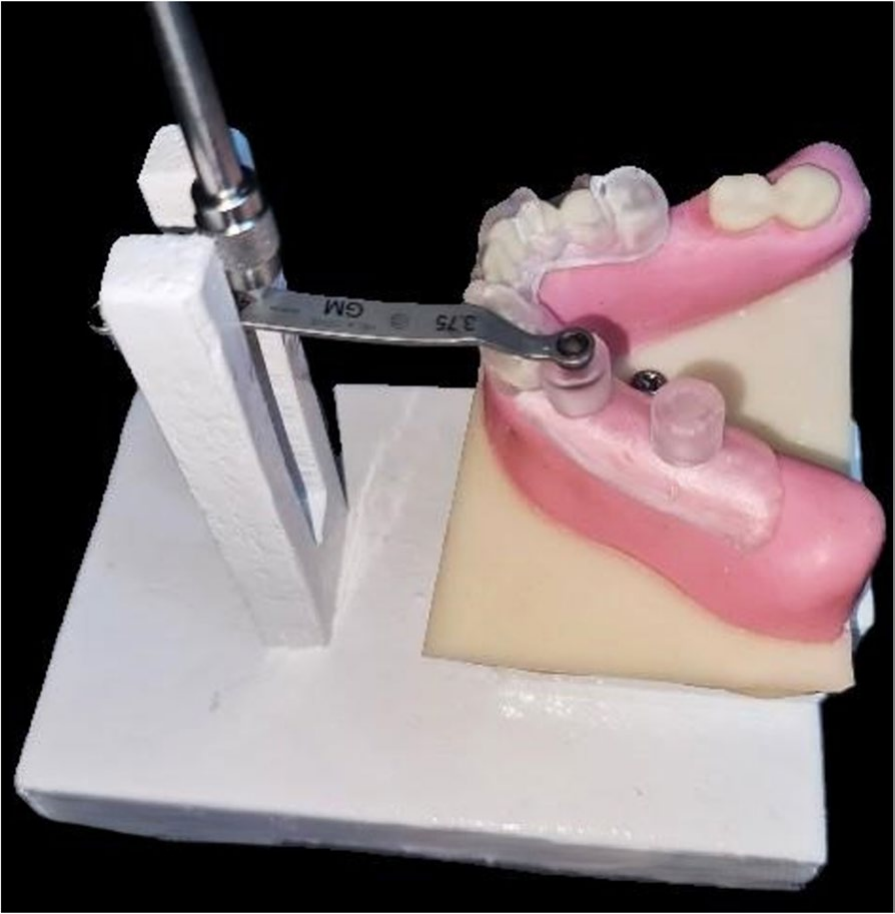
Model with guide on testing platform, during applying force in the sleeve of tooth #34

The current research relied on the use of the testing equipment developed by Kobe et al. [24]. This approach allowed consistent insertion of the dental model in the digital force gauge with a precise 5 cm gap between the crank of the force gauge and the midpoint of the surgical guide's sleeve. The testing platform also featured a slot that allowed the drill handle to be guided while exerting force. Finally, a matching connector was attached to the dental models. This approach made it possible to connect the dental model to the testing platform's model stage (Fig. 2).

The dental models were sprayed with artificial saliva (Xerostom, Biocosmetics) to mimic the clinical environment. The first guide-fitting position on the dental models was recorded using an intraoral scanner (Cerec Omnicam, Sirona, version 4.5.2). Next, a drill handle was attached to the sleeves, and a force gauge (Mxmoonfree 500 N) was manipulated to apply forces of 0.1 N, 1 N, 2.5 N, and 5 N in magnitude. Eight forces were applied to each sleeve, four from the buccal and four from the oral directions (Fig. 2) [24]. An intraoral scanner was used to record the surgical guide displacement while the application of each force, enabling the detection of the deviation under load conditions. This process resulted in a total of 180 scans (20 for the initial fitting of the surgical guide and 160 for the guide deviation following force application).

**Fig. 3:**
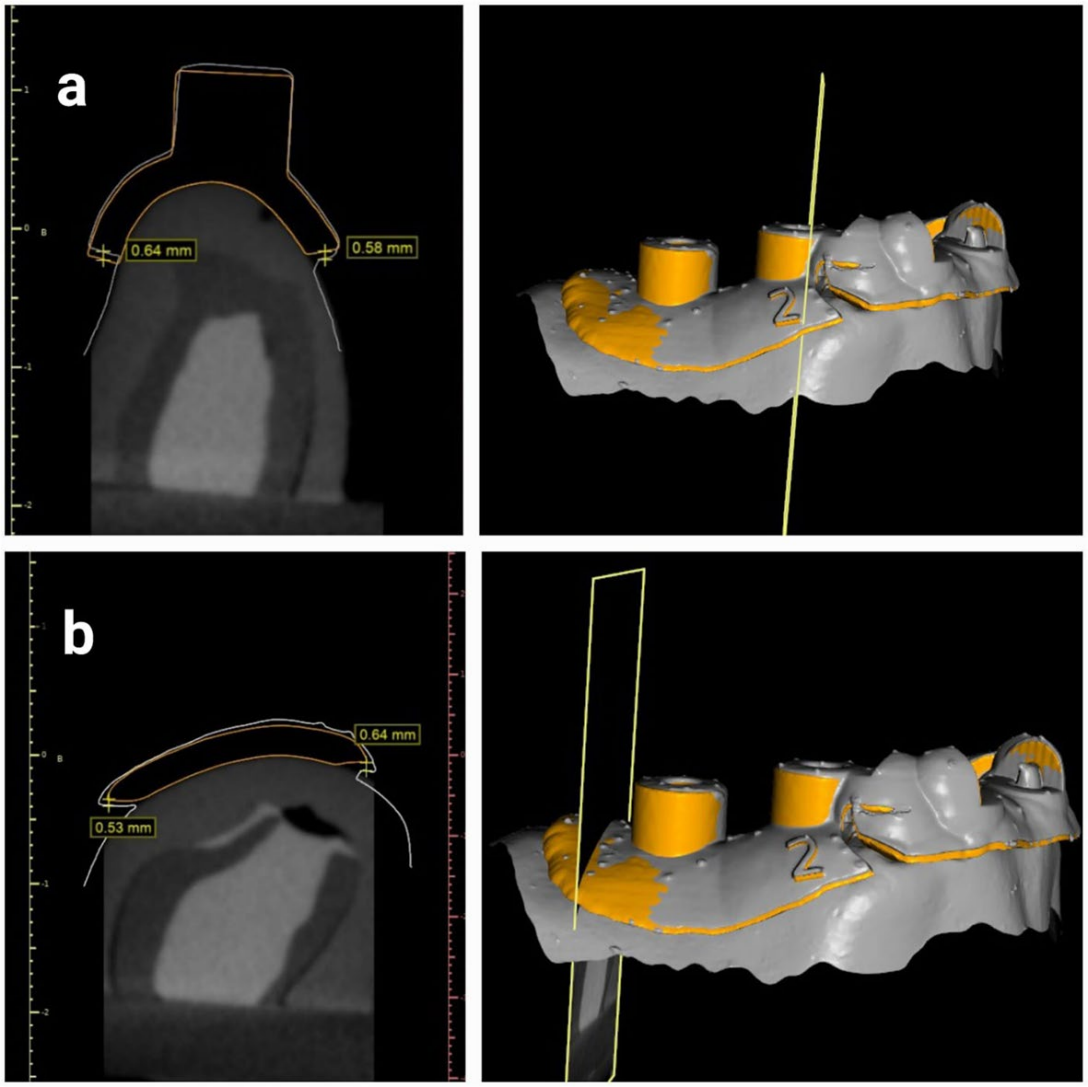
Baseline deviation measurements of the surgical guide. Orange planned surgical guide with retentive design and gray scan of the initial surgical guide position (**a**) mesiobuccally and mesiolingual measurements from sleeves (**b**) distobuccal and distolingual measurements

The distance between four standardized selected reference points on the planned surgical guide library file and the scanned initial position of the surgical guide before the application of force was measured to define the baseline deviation of the guide. The manually selected points were on the mesiobuccal, distobuccal, mesiolingual, and distolingual aspects of the surgical guide sleeves (Fig. 3).

Simulated implants were aligned to the guide orientation hole of the scans, through superimposition with the planned surgical guide using 3-Matic software (3-Matic Research 13.0, Materialise). Initial alignment was performed using manual registration based on three reference points on the surgical guide: one at the sleeve area, one on the anterior aspect, and one on the contralateral side. This was followed by global registration to achieve precise overall superimposition. To ascertain the implant's deviation from the original plan, scans with simulated implants were imported into the planning project in BlueSky software.

**Fig. 4:**
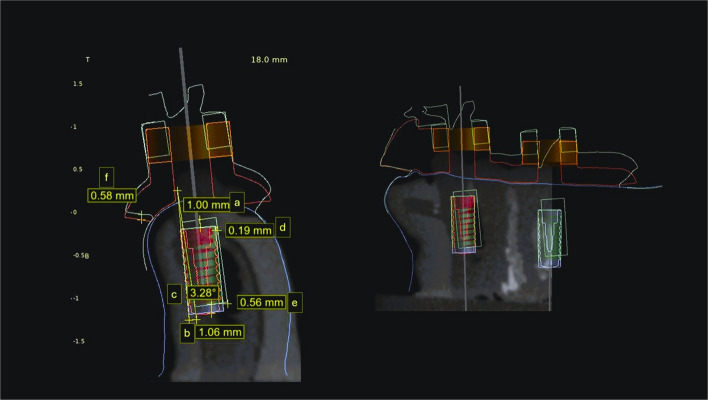
Implant deviation measurement. (**a)** vertical deviation coronal (**b**) vertical deviation apical (**c**) angular deviation (**d**) horizontal deviation coronal (**e**) horizontal deviation apical and (**f**) guide deviation with red planned guide and green guide after 2.5N force application

Superimposition of the preoperative models containing the planned implants with the postoperative scanned models containing the simulated implants was carried out using a surface-based registration method. Initially, three stable anatomical reference points were manually selected on the adjacent teeth (cusp tips and incisal edges) to establish the preliminary alignment. This was followed by an automated best-fit algorithm provided by the software to refine the registration and minimize errors. After registration, deviations between the planned and simulated implant positions were calculated. Three-dimensional measurements at the implant neck, implant apex, and angular implant deviation were used to express the deviation of the implant positions (Fig. 4) [21]. Angular deviation was measured as the angle between the long axes of the planned and simulated implants, depth deviation as the vertical distance between the coronal and apical reference points, and horizontal deviations as the linear distance at both the coronal and apical levels. All measurements were performed within BlueSky software (BlueSky-Plan 4; BlueSky Bio) to ensure consistency.

A single calibrated operator (E.A.E) performed the measurements and was blinded to the magnitude and direction of the applied forces, but not to the guide design. The measurements were recorded manually. Intra-examiner reliability testing was not conducted for the measurement of implant deviation, as all measurements were performed using an automated digital workflow with predefined reference points and standardized superimposition protocols. This minimized operator-dependent variability and ensured consistency across all measurements [24, 34].

The measurements were analyzed by a statistician who was not aware of the groupings. All the data were analyzed using statistical software (SPSS version 27.0). The normality of the study variables was tested. Means and standard deviations (SDs) were calculated for the implant vertical deviation, angular deviation, horizontal deviation coronal, horizontal deviation apical, and guide deviation. The study groups were compared using one-way ANOVA, and then multiple pairwise comparisons with Bonferroni-adjusted significance were carried out. Multivariate ANOVA was used to evaluate the effects of surgical guide design and other variables, with their interactions on deviation measurements.

## Results

Complete displacement of the surgical guide was reported in a retentive design four times, three of which were with forces from the oral direction. In the fixation pin design, complete guide displacement occurred three times, all involving forces from the buccal direction. In total, 306 implants were included in the study: 152 implants were positioned with a retentive design, while 154 were positioned with a fixation design.

The initial baseline deviation of the surgical guide was 0.34 ± 0.19 mm for the retentive design and 0.30 ± 0.14 mm for the fixation design; however, the difference was not statistically significant, as shown in Table 1. The surgical guide and implant deviation measurements are provided in Table 2.

**Table 1:** Initial baseline deviation in surgical guides of two designs

	Design	
	Retentive guide	Fixation guide
N	10	10
Min – Max	0.10—0.65	0.08—0.54
Mean ± Std. Deviation	0.34 ± 0.19	0.30 ± 0.14
SEM	0.06	0.05
95% CI for median	0.21—0.47	0.20—0.40
25th Percentile – 75th Percentile	0.22—0.49	0.22—0.34
*p-value*	*P* = *1.00*	

*n* Number of specimens, *Min-Max* Minimm-Maimum, *CI *Confidence interval, *SEM* Standard error of the mean

Statistically significant *(p<0*.05)

*NS *Statistically not significant *(p*≥0.05)

**Table 2:** Deviation measurements in retentive and fixation design after force application described using Means ± SD

	Retentive design	Fixation design	F	P value
Implant Vertical deviation (mm)	0.99 ± 0.76	0.50 ± 0.34	169.94	< 0.001*
Implant Angular deviation (^o^)	3.60 ± 2.40	2.63 ± 1.25	30.99	< 0.001*
Horizontal deviation apical(mm)	0.97 ± 0.76	0.69 ± 0.48	24.05	< 0.001*
Horizontal deviation coronal (mm)	0.54 ± 0.53	0.36 ± 0.19	26.31	< 0.001*
Guide deviation (mm)	1.02 ± 0.66	0.61 ± 0.28	107.19	< 0.001*

*N* Number of specimens

^*^Statistically significant *(p<0*.05)

*NS* Statistically not significant *(p*≥0.05)

**Fig. 5:**
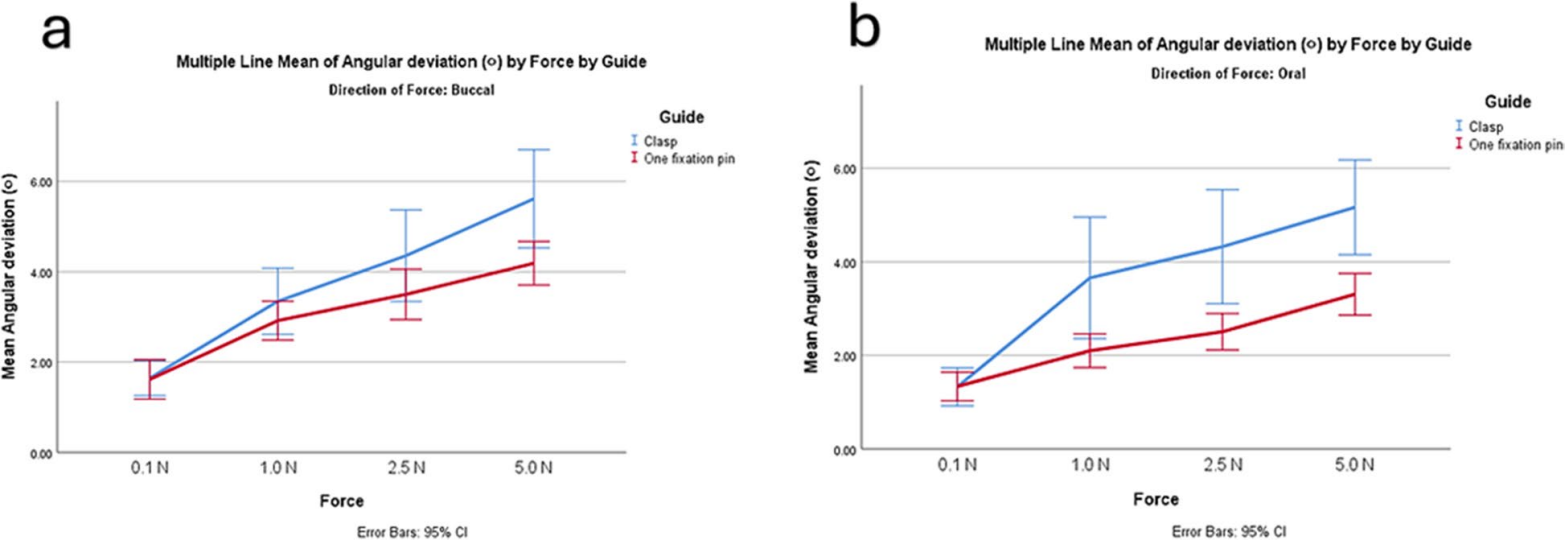
Graph showing angular implant deviation with increasing forces in the two designs. **a** forces from buccal direction, **b** forces from oral direction

**Fig. 6:**
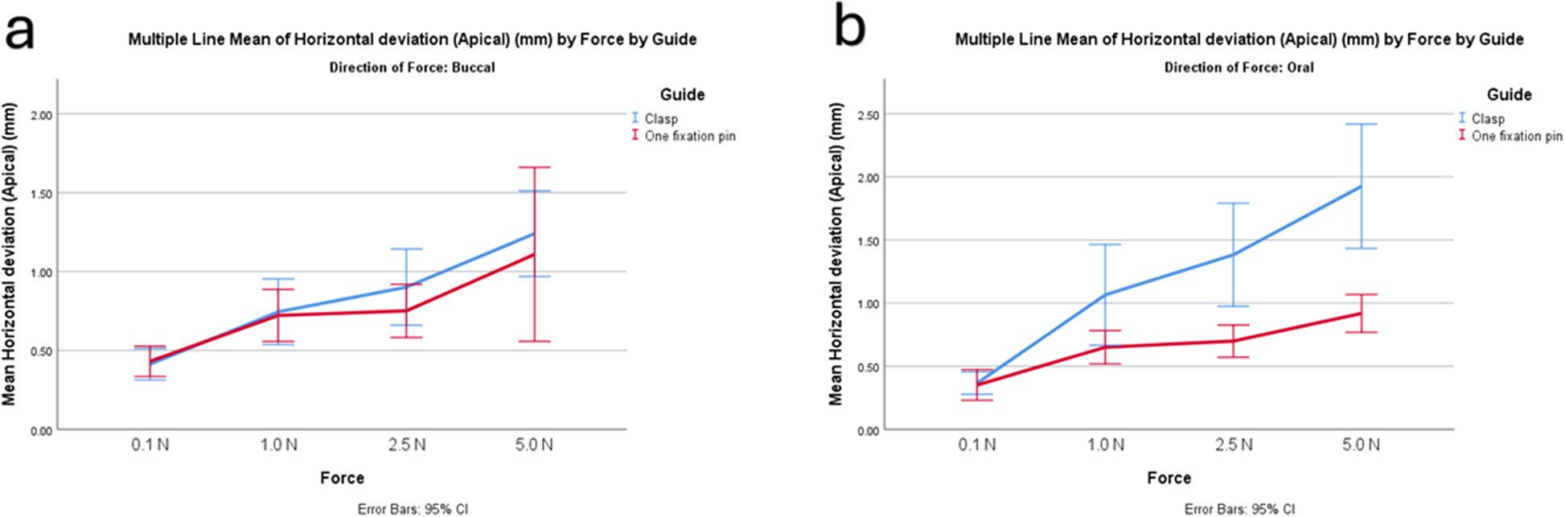
Graph showing implant horizontal deviation with increasing forces in the two designs. **a** forces from buccal direction, **b** forces from oral direction

In all the deviation measurements, the retentive group showed greater deviation compared to the fixation group. Larger angular deviation occurred with increasing force, particularly in the retentive group (Fig. 5). Both designs showed similar horizontal deviation apical with lower forces (Fig. 6). The results of multivariable ANOVA are represented in Table 3. For the implant vertical deviation, the guide design had the largest effect size (*P* < 0.001). For implant angular deviation, the force magnitude had the largest effect (*P* < 0.001, η2 = 0.37, F = 599) followed by the guide design (*P* < 0.001, η2 = 0.1, F = 31).

**Table 3:** Results of ANOVA comparing guide design, force direction, magnitude, and implant location on vertical deviation

	**SS**	**DF**	**MS**	**F**	**Sig**	**Partial Eta Squared**	**Observed Power **^**b**^
Corrected Model	89.67^a^	31	2.89	21.41	** < 0.001**	0.71	1.00
Intercept	180.17	1	180.17	1333.54	** < 0.001**	0.83	1.00
Implant location	9.02	1	9.02	66.75	** < 0.001**	0.20	1.00
Force	13.98	3	4.66	34.49	** < 0.001**	0.27	1.00
Direction of force	19.55	1	19.55	144.68	** < 0.001**	0.35	1.00
Guide	22.96	1	22.96	169.94	** < 0.001**	0.38	1.00
Implant location * Force	1.62	3	0.54	4.00	**0.008**	0.04	0.84
Implant location * Direction of force	3.71	1	3.71	27.48	** < 0.001**	0.09	1.00
Implant location * Guide	0.80	1	0.80	5.94	**0.015**	0.02	0.68
Force * Direction of force	12.55	3	4.18	30.96	** < 0.001**	0.25	1.00
Force * Guide	6.50	3	2.17	16.04	** < 0.001**	0.15	1.00
Direction of force * Guide	5.17	1	5.17	38.27	** < 0.001**	0.12	1.00
Tooth * Force * Direction of force	1.80	3	0.60	4.43	**0.005**	0.05	0.87
Tooth * Force * Guide	0.81	3	0.27	1.99	0.115	0.02	0.51
Tooth * Direction of force * Guide	0.91	1	0.91	6.77	**0.010**	0.02	0.74
Force * Direction of force * Guide	3.47	3	1.16	8.56	** < 0.001**	0.09	0.99
Tooth * Force * Direction of force * Guide	0.64	3	0.21	1.58	0.194	0.02	0.41

*SS* Sum of squares, *DF* Degrees of freedom, *MS* Mean squares

1. R Squared = 0.708 (Adjusted R Squared =.675)

^b^Computed using alpha =.05

Significant effects are indicated in bold

The implant vertical deviation was lessened with forces applied from the buccal direction than from the oral direction (0.52 ± 0.32 mm,1.04 ± 0.79 mm, *P* < 0.001, F = 144.68) and greater with the implant replacing the first molar than the premolar (0.91 ± 0.78 mm, 0.59 ± 0.42 mm, *P* < 0.001, F = 66.75). However, the implant angular deviation was greater with forces applied from the buccal direction (3.40 ± 0.13°, 2.95 ± 0.13°, *P* = 0.013, F = 6.2), while no difference in angular deviation between implants replacing molar and premolar teeth. Larger deviations within oral forces than within buccal forces were detected for implant horizontal coronal deviation (0.55 ± 0.28 mm, 0.37 ± 0.28; *P* < 0.001, F = 19.74) and horizontal apical deviation (0.92 ± 0.043 mm, 0.79 ± 0.043 mm; *P* = 0.034, F = 4.52) (Fig. 5).

## Discussion

Based on the findings of this study, the null hypothesis is rejected. The guide design strongly influenced the vertical deviation of the implants. The fixation pin design led to less displacement of the distal free end surgical guides, with simulated implant deviations decreasing significantly, suggesting a potential advantage in minimizing guide movement during implant site preparation. However, both guide designs demonstrated acceptable simulated implant displacement values [21].

Different forces magnitudes and directions are applied during guided implant surgery, with the highest generated forces reported during the implant osteotomy preparation. It was reported that up to a depth of 4 mm in the mandible, less than 4 N of drilling force was applied. However, upon reaching the lingual cortical bone, the drilling force increased up to 12 N [35]. In Kobe et al. [24]. method and the present study, the forces used were 0.1 N, 1 N, 2.5 N, and 5 N. Therefore, those forces could be comparable to the initial drilling forces.

The use of a sleeveless surgical guide is described in the literature for precise implant positioning [2, 3]. Therefore, this model was implemented in the present study. Furthermore, this sleeveless approach allows all the applied forces in this study to be transferred to the surgical guide directly. In addition, measuring implant deviation was performed with BlueSky planning software using a cross-sectional view rather than 3-Matic superimposition software. In contrast, in the Kobe et al. [24] study, deviation measurements and superimposition were performed with one software package (GOM Inspect software).

The use of a polyurethane-based material to simulate soft tissue in this study was selected because it is commonly applied in in vitro implant research, providing ease of manipulation, dimensional stability, and standardized mechanical properties across samples. These features help reduce variability and ensure reproducibility of measurements [32]. Nevertheless, its biomechanical behavior does not fully replicate the viscoelastic characteristics of human oral mucosa, particularly in terms of resilience and compressibility. This difference should be considered when extrapolating the present findings to clinical scenarios.

In the present study, the baseline deviation of the surgical guides was slightly higher in the retentive design group than in the fixation design group, but the difference was not statistically significant. The fitting of the retentive guide could be affected by the reduced guide-teeth offset. Moreover, Kobe et al. [24] reported similar findings. In the literature, an offset between 0.1 and 0.2 mm is typically advised to compensate for CBCT, optical scanning, and superimposition inaccuracies [5, 9, 10, 36]. The findings of the current study imply the importance of this recommended offset.

Based on the findings of the current study, the fixation design was superior to the retentive surgical guide design in terms of stability and resulting implant deviation. This result was different than that of Kobe et al. [24] They applied a retentive design in bilateral tooth-supported guides with a single missing tooth, while this study applied their design in a unilateral tooth mucosa-supported guide. The increased guide extension in the current study might compromise guide accuracy. In addition, the retentive design was compared to the guide with another means of retention, which is dissimilar to the approach used by Kobe et al. [24], who compared the retentive design against a conventional guide with four teeth extension.

Furthermore, Kobe et al. [24] excluded the 5 N force group due to the increased number of dislodgements. In the present study, complete displacement of the surgical guides occurred 5 times out of 40 within the application of a 5 N force. In addition, as mentioned previously, the drilling forces could clinically exceed 4 N; thus, in this study, the 5 N force group was not excluded. Moreover, both surgical guide designs showed comparable results in resisting smaller forces. Accordingly, this could explain the better results of the retentive design in Kobe et al. [24].

The accuracy of surgical guide fabrication is influenced by several cumulative steps in the digital workflow, including intraoral or extraoral scanning, radiographic imaging, data alignment, guide design, and 3D printing. Among these, intraoral scanning has been identified as a critical source of potential error, especially in partially edentulous arches or long-span scans. Intraoral scanners are susceptible to inaccuracies due to limited soft tissue retraction, saliva, and stitching artifacts, particularly in the posterior mandible [5, 9, 36]. These deviations can affect the trueness of the digital model and subsequently impact guide fit and implant positioning. In this study, although an extraoral scanner was used, the clinical translation of these results would require caution, as intraoral scanning introduces additional variability. Therefore, the surgical guide’s baseline deviation and implant positional accuracy may differ when the guide is fabricated from intraoral scans versus model scans. Studies by Abduo et al. [10] and Jayaratne et al. [9] have reported that even small scan misalignments or segmentation errors can translate to clinically relevant deviations in implant placement. Thus, optimizing each digital step, particularly intraoral scanning, is crucial for improving the final accuracy of implant-guided surgery.

The resilience and compressibility of the mucosa present a challenge for achieving stable support in tooth-mucosa-supported surgical guides. Studies have shown that mucosal deformation under load can lead to positional deviations of the guide, especially in edentulous or distal extension cases [12, 15]. This highlights the importance of minimizing reliance on soft tissue and incorporating rigid retention features such as fixation pins or tooth undercuts.

Although the present study did not involve actual implant placement, the simulated deviations observed, particularly in the retentive design (angular deviation of 3.60 ± 2.40° and vertical deviation of 0.99 ± 0.76 mm), fall within the range reported in the literature [8].

Forces from oral direction caused larger implants' vertical and horizontal deviations. suggesting that the direction of applied loading can significantly influence implant accuracy. The extension of the surgical guide to the contralateral side possibly helped in resisting deviations from buccal forces. However, oral forces depend on distal teeth for vertical stability, and since distal extension models were used, they exhibited more vertical deviation. This also explains the increased resistance to dislodgement when fixation pins are placed in the distal extension of the guide with oral forces. Therefore, caution should be exercised regarding forces applied from the oral direction in free-end saddle cases, particularly when implant vertical deviation is critical, such as in proximity to anatomical landmarks. Furthermore, placing a fixation pin in the distal part of the edentulous area is strongly recommended in such cases.

Regarding the implant site, this study found larger deviations in simulated molar placements compared to premolars. This has also been observed in clinical settings, as reported by Pessoa et al. [18] in patients treated with a unilateral tooth-supported guide. Clinically, that could increase the risk of encroachment on vital anatomical structures, particularly the mandibular canal and maxillary sinus, underscoring the need for meticulous planning of implant placement in the molar area.

Furthermore, guide design, implant site, force magnitude, and force direction all have a significant impact on implant vertical deviation, according to the multivariate analysis conducted for this study. However, the only factor that significantly affected the implant angular deviation was the force magnitude. Therefore, limiting the force created on surgical guides during surgery is crucial, even though surgical guide design is important.

The limitations of the current study include the force used on the drill handle, which may not accurately reflect the forces used to prepare the implant bed. Moreover, the chosen force measurement device with a range of 0–500 N, as a device with a narrower range (e.g., 0–10 N) would provide higher sensitivity, better accuracy, and improved reproducibility. One limitation of this study is the use of polyurethane-based material to simulate soft tissue. While commonly used in in vitro studies for its elastic properties and standardization, it does not perfectly mimic the biomechanical characteristics of human mucosa. Owing to the in vitro nature of the study, numerous other elements were overlooked, including the surgeon's position, mouth opening, guide location inside the dental arch, and surgical access. Furthermore, implant placement was performed simulated. Therefore, other clinical studies evaluating the surgical guide designs mentioned in this study are recommended, along with different guide extensions.

Changing the predetermined parameters in the planning software and creating a clasp-like part for the fabrication of retentive surgical guide designs requires additional steps, which could complicate the design of surgical guides. Therefore, the fixation of surgical guides in unilateral distal extension bases is preferred. Furthermore, care should be taken when applying forces to surgical guides, especially when implants replace mandibular molar teeth.

## Conclusion

Based on the findings of this in vitro study, the following conclusions were drawn:

1. Incorporating retention features into surgical guides significantly affects guide stability and simulated implant displacement.
2. The use of fixation pins in the surgical guide design demonstrated superior accuracy compared to the retentive design.
3. The unilateral tooth-supported retentive surgical guide achieved acceptable accuracy in simulated implant placement.

## Data Availability

The datasets created or analyzed during the current study are not publicly available but are available from the corresponding author upon reasonable request.
